# Tips and Tricks in Thoracic Radiology for Beginners: A Findings-Based Approach

**DOI:** 10.3390/tomography9030095

**Published:** 2023-06-14

**Authors:** Alessandra Borgheresi, Andrea Agostini, Luca Pierpaoli, Alessandra Bruno, Tommaso Valeri, Ginevra Danti, Eleonora Bicci, Michela Gabelloni, Federica De Muzio, Maria Chiara Brunese, Federico Bruno, Pierpaolo Palumbo, Roberta Fusco, Vincenza Granata, Nicoletta Gandolfo, Vittorio Miele, Antonio Barile, Andrea Giovagnoni

**Affiliations:** 1 Department of Clinical, Special and Dental Sciences, University Politecnica delle Marche, Via Tronto 10/a, 60126 Ancona, Italy; 2 Department of Radiology, University Hospital “Azienda Ospedaliero Universitaria delle Marche”, Via Conca 71, 60126 Ancona, Italy; 3 Italian Society of Medical and Interventional Radiology (SIRM), SIRM Foundation, 20122 Milan, Italy; 4 School of Radiology, University Politecnica delle Marche, Via Tronto 10/a, 60126 Ancona, Italy; 5 Department of Radiology, Azienda Ospedaliero-Universitaria Careggi, 50134 Florence, Italy; 6 Nuclear Medicine Unit, Department of Translational Research, University of Pisa, 56126 Pisa, Italy; 7 Department of Medicine and Health Sciences V. Tiberio, University of Molise, 86100 Campobasso, Italy; 8 Department of Diagnostic Imaging, Area of Cardiovascular and Interventional Imaging, Abruzzo Health, Unit 1, 67100 L’Aquila, Italy; 9 Medical Oncology Division, Igea SpA, 80013 Naples, Italy; 10 Division of Radiology, Istituto Nazionale Tumori IRCCS Fondazione Pascale—IRCCS di Napoli, 80131 Naples, Italy; 11 Diagnostic Imaging Department, Villa Scassi Hospital-ASL 3, 16149 Genoa, Italy; 12 Department of Biotechnological and Applied Clinical Sciences, University of L’Aquila, 67100 L’Aquila, Italy

**Keywords:** chest, lung, computed tomography, lung cancer, solitary pulmonary nodule, diffuse lung disease, radiologist in training

## Abstract

This review has the purpose of illustrating schematically and comprehensively the key concepts for the beginner who approaches chest radiology for the first time. The approach to thoracic imaging may be challenging for the beginner due to the wide spectrum of diseases, their overlap, and the complexity of radiological findings. The first step consists of the proper assessment of the basic imaging findings. This review is divided into three main districts (mediastinum, pleura, focal and diffuse diseases of the lung parenchyma): the main findings will be discussed in a clinical scenario. Radiological tips and tricks, and relative clinical background, will be provided to orient the beginner toward the differential diagnoses of the main thoracic diseases.

## 1. Introduction

Thoracic imaging is fundamental for the assessment of lung parenchyma, the pleural district, and the mediastinal structures [1]. In most cases, chest X-ray (CXR) and computed tomography (CT) provide diagnostic information with a high degree of confidence [2]. The association of typical radiological findings with clinical or laboratory data leads to the right diagnosis. On the other hand, some diagnoses are challenging and can be achieved only with surgical or biopsy specimens. These cases require a multidisciplinary approach for correct management, including experienced pneumologists, chest radiologists, pathologists, and thoracic surgeons [3,4].

The purpose of this review is to provide the basics for the diagnostic approach to pulmonary, pleural, and mediastinal diseases, together with “tips and tricks” that can be helpful for the beginner. The paper will be divided into three main sections (i.e., differential diagnosis of mediastinal masses, differential diagnosis of pleural lesions, and differential diagnosis of parenchymal diseases) based on the main anatomical structures of the chest to provide a diagnostic approach as plain as possible for residents and beginners. Furthermore, the section on the mediastinum is divided into the main anatomical compartments since it is relevant for the differential diagnosis of mediastinal lesions. The diagnostic approach of pleural diseases considers neoplastic and non-neoplastic or tumorlike lesions. The differential diagnosis of pulmonary diseases is divided into focal or diffuse lesions. The focal pulmonary lesions are divided following the basic findings on CXR or CT (e.g., shape, margins, density…), while the diffuse lung diseases are divided following the main patterns on CT.

## 2. Differential Diagnosis of Mediastinal Masses

The differential diagnosis of mediastinal masses covers a broad variety of pathologic entities; a widely used method to start the diagnostic process is the localization of the pathological process within a mediastinal compartment [4,5,6]. The International Thymic Malignancy Interest Group (ITMIG) developed a structured approach to the differential diagnosis of mediastinal masses based on a cross-sectional imaging classification that identifies three mediastinal compartments: prevascular (anterior), visceral (middle), and paravertebral (posterior) [7,8]. This classification is also widely used in conventional radiology; the combination of the silhouette sign and the knowledge of mediastinal reflections in CXR can help to correctly identify the location of a mass [9]. Table 1 summarizes the main anatomical landmarks and contents of the mediastinal compartments and the main findings for the correct location of a mediastinal mass at CXR [10,11,12,13,14].

Once the mass is identified on the CXR, then CT and MRI provide more detailed information about the anatomical relationship of the tumor, with additional but fundamental data if contrast material is administered. Moreover, CT and MRI allow for the evaluation of lesion composition (cystic nature, presence of calcifications, or fat content) [15,16,17,18]. Table 2 summarizes the main mediastinal lesions sorted by location and composition with the relative frequencies [19].

Mediastinal masses are quite rare; however, slightly more than half of them are in the anterior mediastinum, and the others are almost equally divided between the middle and posterior compartments [7].

In the following sections, we will describe the mediastinal lesions that present characteristic features at radiology. In most cases, the integration between the imaging findings and clinical information lead to a confident diagnosis; however, the multidisciplinary approach is necessary for more complex cases [4].

### 2.1. Prevascular Compartment

#### 2.1.1. Fat-Containing Lesions

The presence of macroscopic intralesional fat can be confidently assessed on CT when Hounsfield Unit (HU) values between −40 and −120 are measured, or on MRI by any sequence including fat suppression or chemical shift imaging [5,20]. One of the most overlooked benign lesions on chest CXR is the *epicardial fat pad*, which appears as a para-cardiac mass in contact with the diaphragm. On CT, it can be easily assessed since it appears as very homogeneous fat tissue, usually at the cardiophrenic angle, without any encapsulation, typically more prominent in obese patients [21]. *Mature teratoma* commonly affects young patients; it represents 25% of the lesions of the anterior compartment in ages 10–19, 10–15% in ages 20–49, and <5% for age > 50 in both men and women [17]. It is a highly characteristic lesion on imaging, showing a heterogeneous content for the presence of intralesional fat (50% of cases), and varying amounts of fluids, soft tissues, and calcifications (including bone and toothlike elements) [20]. The presence of a fat–fluid level is highly specific for this benign lesion; however, it is a rare finding [17,22]. Another fat-containing lesion is the *thymolipoma*; it accounts for less than <5% of prevascular masses in all age groups without sex predilection [6,17]. This benign tumor classically appears as a large (mean 20 cm in size), encapsulated mass located in a cardiophrenic angle, and it is mostly composed of macroscopic fat (up to 95%) with scattered regions of solid tissue and fibrous septa [20,23,24]. Some useful tips for the confirmation of the diagnosis are the demonstration of a direct connection with the thymus (best visualized on MRI) and the characteristics morphological changes at different decubitus positions of the patient [16,18,25]. Mass effect symptoms may be present; rare cases of associations between thymolipomas and myasthenia gravis, Grave’s disease, and hematological disorders have been reported [26]. *Lipoma* represents 2% of all the anterior mediastinal masses and it is a well-defined, encapsulated mass, predominantly composed of homogeneous fat and with a small amount of soft tissue and blood vessels [17]. However, the presence of macroscopic fat should not be univocally associated to benignity; the presence of aggressive features, including a greater proportion of soft-tissue components, local invasion, and lymphadenopathy, should always be investigated to exclude a liposarcoma, which is a very rare lesion but is composed predominantly of fat [20,27,28]. A rare fat-containing lesion is the *Morgagni hernia*; it contains omental fat that has herniated through the diaphragm into the thorax via the foramen of Morgagni and usually contains bowels and gas [6,20]. Morgagni hernias typically occur in adults and may be associated with obesity, trauma, or other causes of increased intra-abdominal pressure [23,29].

#### 2.1.2. Cystic Lesions

**Figure 1:**
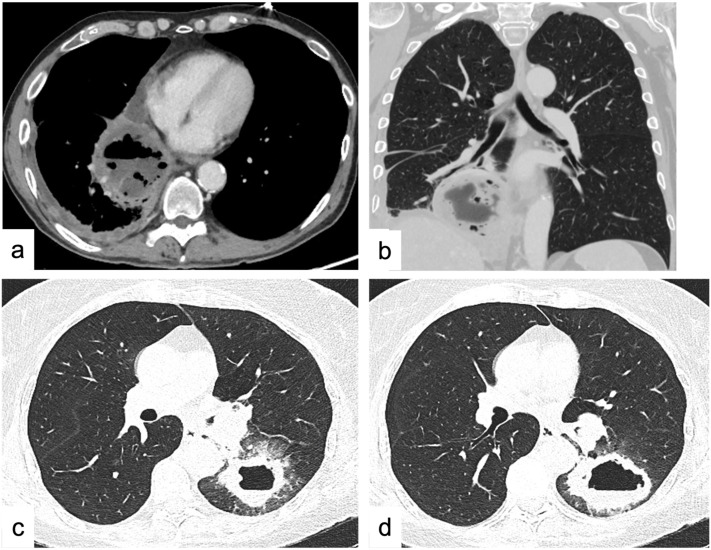
Squamocellular lung cancer vs. lung abscess. Axial CT scan (soft-tissue window) (**a**) and coronal view (lung window) (**b**) in a 70-year-old male patient with fever, cough, and chest pain for a few weeks, with laboratory tests showing infection, and unresponsive to first-line antibiotic therapy. The presence of inner air–fluid level, rim contrast enhancement, and clinical finding are suggestive for lung abscess. Axial CT scans (lung window) (**c**,**d**) in a 66-year-old male patient: the presence of air–fluid inner level may be indicative of lung abscess, but the absence of clinical evidence of infection, thick walls, ground-glass opacities in the surrounding pulmonary parenchyma, and small satellite pulmonary nodules are suggestive for squamocellular lung cancer.

Typically, a cystic lesion is a rounded/oval homogeneous mass with a thin wall and HU values ranging between 0 and 20 HU without sign of enhancement or infiltration of the adjacent structures [20]. The confirmation of the purely cystic nature should be placed by MRI where the cyst typically shows high signal intensity on T2-weighted images [30,31]. Cystic lesions placed near the thymic bed that do not demonstrate soft-tissue components or internal septa are highly characteristic of a thymic cyst; similarly, if the cyst is placed in one of the cardiophrenic angles (mostly the right one) it can be confidently diagnosed as a *pericardial cyst* [32,33]. Thymic cysts are mostly due to an inflammation or iatrogenic processes (surgery, radiation therapy, or chemotherapy) and may demonstrate the presence of hemorrhagic or proteinaceous components that are better demonstrated on MRI [30,31]. MRI also allows for the identification of the internal soft-tissue components and/or internal septa [6,34]. In these cases, the differential diagnosis includes multilocular thymic cysts, cystic thymoma, lymphangioma, or cystic teratoma. The presence of *cystic thymoma* should be strongly considered if the patient also demonstrates symptoms related to myasthenia gravis or other paraneoplastic syndromes, especially if >40 years of age. Each of the above-described cystic lesions represents <5% of the anterior mediastinal lesions [17]. *Lymphangioma* is a very rare benign congenital malformation consisting of a large multilocular cystic lesion (spongelike) that may extend into the neck, axilla, or chest wall, and it is usually discovered during the first two years of life [33,35,36]. *Cystic teratoma* is a variant of the mature teratoma where the fatty component is predominantly or entirely replaced by a unilocular or multilocular thin-walled cystic mass. Furthermore, some solid tumors (lymphomas) or abscesses can demonstrate cystic degenerative changes (Figure 1); however, in such cases, laboratory tests, patient history (previous median sternotomy, esophageal perforation, or recent head/neck infections), and ancillary features (air bubbles) are helpful clues for the diagnosis [33].

#### 2.1.3. Soft-Tissue Enhancing Masses

*Mediastinal goiter* is one of the most common lesions of the prevascular compartment (20–40%, age > 40) and has highly characteristic features on imaging. It is heterogeneous and characteristically hyperdense (with HU of 70–85 due to the presence of iodine), it demonstrates intense and prolonged enhancement (>2 min) after administration of IV contrast, and it is often connected to the thyroid gland [37]. It usually demonstrates cystic changes; calcifications may be present. Sometimes, a definitive connection with the thyroid gland cannot be identified; nevertheless, when they are separate, they often demonstrate similar imaging features, and the diagnosis can be confidently achieved [38]. It is fundamental to always consider the signs of malignancy, loss of distinct mediastinal fascial planes, or local lymphadenopathy, which can provide the suspect of thyroid carcinoma [1,16].

**Figure 2:**
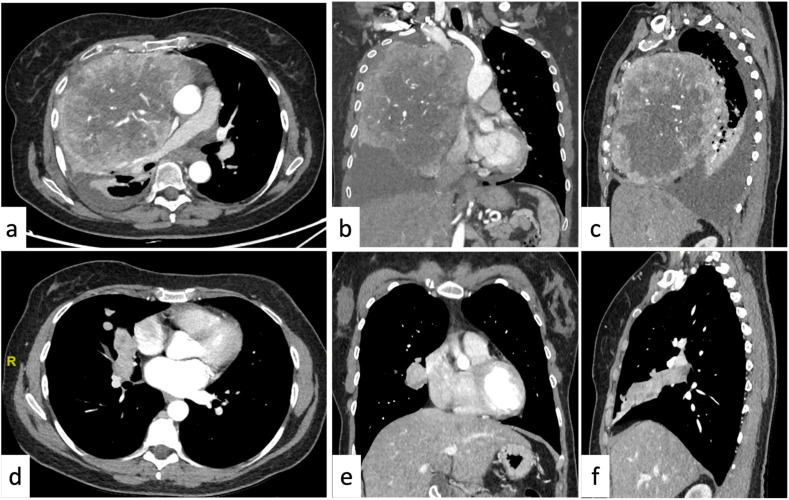
Mediastinal neuroendocrine tumor (NET) vs. lung carcinoid. (**a**–**c**) Mediastinal neuroendocrine tumor (NET). 40-year-old male patient with a sensation of chest weight and dyspnea. Contrast-enhanced axial CT scans (**a**) with coronal (**b**) and sagittal (**c**) reconstruction show a voluminous vascularized solid mass, markedly inhomogeneous for the presence of necrotic tissue, occupying most of the right hemithorax, probably originating from the anterior mediastinum; it determines dislocation, compression, and infiltration of the vascular structures of the mediastinum, thus like pleural planes. Ipsilateral pleural effusion and overlying pulmonary parenchyma atelectasis are associated. (**d**–**f**) Similar CT features are seen in a 44-year-old female. Contrast-enhanced CT (**d**–**f**): it shows a solid nodule in the right ilo-parahilar site of the middle lobe, with inhomogeneous post-contrastographic enhancement. This lesion appears in proximity to the lateral wall of the right atrium (without clear signs of infiltration). These characteristics are suspicious for malignant nature; the biopsy confirmed the diagnosis of typical pulmonary carcinoid.

*Thymoma* should be considered in cases of homogeneous or slightly heterogeneous anterior mediastinal mass in patients >40 years of age since it represents about 50% of the anterior compartment lesions in this age range. If it occurs in association with symptoms of myasthenia gravis or other paraneoplastic syndromes, the diagnosis can be determined confidently [17,39,40]. Typically, lymphadenopathy is not present, but thymomas may present signs of local invasiveness for pleural and/or pericardial spread in advanced stages [22]. Conversely, other thymic epithelial neoplasms should be suspected (*thymic carcinoma* or *carcinoid*) in this age range in case of a large heterogeneous mass with calcification, necrotic or cystic component, irregular contour, and enhancement paired with signs of aggressive behavior (lymphadenopathy, pleural effusion, and sign of local invasiveness). Carcinoid tumors typically show a vivid arterial enhancement and may be associated with endocrine neoplasia (MEN) type 1 [10]. The suspect can be further confirmed on 18 F-FDG PET/CT, as they typically demonstrate higher FDG uptake [41,42,43,44]; however, thymic epithelial neoplasms are rare conditions since they represent about 5% in >40 years of age (Figure 2) [17].

In the setting of a soft-tissue mildly enhancing mass in the mediastinum, if lymphadenopathy is present, *lymphoma* should be considered. Additionally, far from thymic epithelial neoplasms and germ cell tumors, lymphomas show an infiltrative nature that encircles but “respects” the great vessels without invasion. Lymphomas represent the most common mediastinal lesion in <40 years of age, thus, when associated with “B” symptoms (i.e., fever, weight loss, and night sweats), it is possible to place quite confidently the diagnosis. If these findings are combined with pleural effusion and elevated serum levels of lactate dehydrogenase, then *lymphoblastic non-Hodgkin lymphoma* should be considered.

Another lesion to be considered in the differential diagnosis for a large, lobular homogeneous anterior mediastinal mass in a young man 10–39 years of age is *seminoma* [45]. This may be difficult to be differentiated from lymphoma on imaging; however, it is usually lobulated or irregular in shape and the presence of distant metastases (usually in lungs) and slightly elevated serum β-HCG (10% of cases) could be the tiebreaker for seminoma diagnosis [46]. The serum lactate dehydrogenase levels are usually elevated, as in many lymphomas [47,48]. In the case of a heterogeneous anterior mediastinal mass in a patient <40 years old with lung metastases, nonseminomatous germ cell tumors (NSGCTs) should be included in the differential diagnosis [17,49]. Markedly elevated serum α-FP or β-HCG levels are present in 90% of patients and are pathognomonic for this diagnosis [17,34,47,49,50,51,52].

*Thymic hyperplasia* is an uncommon condition that usually manifests as a uniform enlargement of the thymus in young patients or at age > 40 without evidence of a focal mass. In true hyperplasia (or “rebound hyperplasia”), the patient usually reports exposure to stressors (i.e., chemotherapy, radiation therapy, corticosteroids, burns, injuries), and the thymus can demonstrate an increase in size > 50% over the baseline [53]. In thymic lymphoid (follicular) hyperplasia, the patient demonstrates underlying immunological diseases (e.g., myasthenia gravis, hyperthyroidism, collagen vascular diseases, or human immunodeficiency virus (HIV) infection) [17]. Unfortunately, in some cases, a nodular or bulky aspect can be present, and the differential diagnosis with a thymic epithelial tumor, lymphoma, or other soft-tissue neoplasms can be difficult. In these cases, a 3-month follow-up CT should be considered. Another strategy includes an MRI evaluation with in-phase and out-phase gradient echo sequence to assess the type 2 chemical shift artifact [15,54,55]. The fat interspersed within the hyperplastic thymic parenchyma typically demonstrates a signal loss in out-phase images; conversely, thymic epithelial neoplasms, lymphoma, and other soft-tissue malignancies do not demonstrate this artifact [15,54,55].

*Parathyroid adenomas (ectopic)* are usually small in size and have a nonspecific appearance, but they should be suspected if the patient is affected by hyperparathyroidism (elevated serum levels of calcium and parathyroid hormone) [6]. They manifest as a hypervascular, mediastinal lesion with washout of contrast material in the delayed phase; if doubt persists, they can be confidently characterized with Technetium-99 sestamibi single-photon emission CT scans [6,50].

### 2.2. Middle Compartment

#### 2.2.1. Cystic Lesions

A simple cystic lesion (as described above) of this compartment is compatible with a benign duplication cyst, usually bronchogenic or esophageal. *Bronchogenic cysts* are commonly located in the subcarinal area (52%) or, less commonly, in the right paratracheal region (19%); they may involve any mediastinal compartment but typically are in the middle one [33]. Intrinsic calcifications and hemorrhagic or proteinaceous components may occur [33]. The presence of intralesional air is uncommon; it suggests secondary infection and/or communication with the tracheobronchial tree [16,56]. *Esophageal duplication cysts* show similar imaging features; however, they could show thicker walls and are located adjacent to the esophagus or in association with the esophageal wall itself [33]. In this case, the presence of a heterogenous content can be due to ectopic gastric mucosa (50% of cases) which is specifically detected on ^99m^Tc sodium pertechnetate scans [57].

#### 2.2.2. Soft-Tissue Enhancing Masses

The first aspect to be evaluated is the clinical history of the patient looking for the possibility of *metastatic lymphadenopathy* from various primary malignancies (e.g., renal cell and thyroid neoplasms, melanoma, choriocarcinoma, and sarcoma). Furthermore, if the lesion is strictly related to the esophagus, consider *esophageal cancer*, particularly if the wall thickening is focal instead of homogeneous; the diagnosis should be confirmed by endoscopy. The presence of a homogenous, high-enhancing mass is compatible with *paragangliomas* or *extra-adrenal pheochromocytomas*. These lesions can be “functional” and secrete catecholamines; however, most of them are “non-functional” [58,59]. MRI can confirm the suspect if a mass with intermediate signal intensity on T1-weighted images and high signal intensity on T2-weighted images is demonstrated; iodine 123 (^123^I) metaiodobenzylguanidine (MIBG) scintigraphy can help to support the diagnosis [58,59]. A cardiac mass is a rare condition, primary tumors are even more rare, and metastatic diseases should be always excluded first. There are many kinds of cardiac neoplasms; it is important to note that due to their complexity, these lesions should always be properly assessed with advanced imaging (electrocardiographically (ECG) gated cardiac CT, cardiac MR imaging, and/or echocardiography) for adequate characterization [60].

### 2.3. Posterior Compartment

#### 2.3.1. Cystic Lesions

*Intrathoracic meningocele* should be suspected if a cystic lesion in the paravertebral mediastinum is associated with neurofibromatosis or vertebral primary (e.g., hemivertebrae, butterfly vertebra, spina bifida) or acquired (e.g., trauma) abnormalities [6,33,61]. Another cystic lesion with paravertebral location and association with vertebral anomalies is the *neurenteric cyst*. The *neurenteric cyst* is a very rare condition, and the differential diagnosis with the meningocele can be performed by the intraspinal injection of contrast material that will reveal the filling of the meningocele [6,23,61]. *Pancreatic pseudocysts* or *mediastinal abscesses* are cystic masses that usually demonstrate an enhancing wall and heterogenous content because of blood and necrotic material; in such cases, the clinical context permits a definitive diagnosis [62,63]. The extension of a pancreatic pseudocyst into the mediastinum is uncommon; however, the clinical setting of pancreatitis or the presence of similar lesions in the abdomen can support the diagnosis of intrathoracic extension of a pancreatic pseudocyst [63,64]. Conversely, a mediastinal abscess should be suspected after surgery or esophageal perforation or in the setting of infection in the adjacent thorax in a patient with clinical symptoms (e.g., fever) [62].

The posterior mediastinal compartment also includes the thoracic spine, with all the related pathology, such as spinal infections (significant risk factors include diabetes, autoimmune diseases, malignancy, immunosuppression, and intravenous drug use) and primary osseous tumors.

#### 2.3.2. Soft-Tissue Enhancing Masses

Most of the lesions of this compartment are neoplasms of neurogenic origin, and 70–80% of them are benign and represent 20% of all the mediastinal neoplasms in adults [65]. *Neurogenic neoplasms* are characteristically smooth, round, or oval masses located in the paravertebral region and mostly consist of benign peripheral nerve sheath tumors (e.g., schwannoma or neurofibroma) which usually show the classical dumbbell morphology and communication with the spinal canal [65]. Benign, pressure erosion of adjacent ribs or vertebrae, and enlargement of the neural foramina can be present, and this should not be confused with a malignant behavior. A better definition can be obtained with MRI, which demonstrates the extent of intraspinal/extradural extension and the “fascicular sign”, typical for schwannomas, and the “target sign”, more characteristic of neurofibromas [66,67]. However, it must be pointed out that neurofibromas can have malignant transformation; this risk is higher in patients affected by type 1 neurofibromatosis. Malignant transformation of a peripheral nerve sheath tumor should be suspected in case of size increase, heterogeneity, invasiveness, and high FDG uptake at PET/CT [68,69].

*Extramedullary hematopoiesis* should be considered in the setting of a hematologic disorder resulting in bone marrow replacement (myelofibrosis or chronic myelogenous leukemia) or hemolytic anemia (thalassemia, sickle cell anemia, or hereditary spherocytosis) [23,70]. The masses are typically adjacent to thoracic vertebrae and ribs and show vivid enhancement; in the case of proven long-standing lesions, they demonstrate heterogeneous attenuation because of the iron deposition and fat infiltration [20,71]. However, if such underlying disorders are unknown, ^99m^Tc sulfur colloid bone marrow scan and SPECT/CT bone marrow scan may noninvasively confirm the presence of functioning hematopoietic tissue, avoiding unnecessary biopsy [72,73,74].

### 2.4. More than One Mediastinal Compartment

*Lymphadenopathy* is the most common cause of anterior and middle mediastinal masses: it is usually secondary to many pathological processes such as lymphoproliferative disorders (Hodgkin and Non-Hodgkin lymphoma), sarcoidosis, inflammatory or infective conditions, and metastases. It is possible to differentiate between neoplastic and granulomatous processes by morphology. A detailed review of all the conditions and imaging features related to lymphadenopathy is beyond the scope of this paper; however, some basic tips will be discussed in the following section [16,75].

Once the lymph node is localized (the International Association for the Study of Lung Cancer (IASLC) map is recommended), the term *lymphadenopathy* is usually restricted to enlargement, due to any cause, of the lymph nodes [76,77,78]. However, since the dimensional criteria themselves lack specificity for the distinction between normal or pathological conditions, other morphological features must be evaluated [79].

For the dimensional assessment of lymph nodes, the measurement of the short axis is recommended [80]. In general, mediastinal lymph nodes with short axes up to 12 mm and hilar lymph nodes with short axes up to 3 mm are considered benign [79,81,82]. Moreover, it must be pointed out that nodal enlargement does not always represent a pathological condition. The sarcoid-like reactions are temporary, immune-related adverse events to different drugs (e.g., immunotherapy, interferon-α, highly active antiretrovirals) where patients develop mediastinal lymph node enlargement. This condition must be considered in oncological patients in immunotherapy since it can simulate a disease progression. In doubtful cases, a 4-week follow-up CT is warranted [78,83,84].

The other CT features to be evaluated include the increase in number or changes in attenuation (calcific, fatty, hypodense, enhancing). The integration of these findings with the clinical data is helpful to orient the differential diagnosis [77,78].

While the presence of fat or calcification should be assessed on basal acquisitions (or on spectral data of dual-energy datasets), the other features require the administration of contrast material [78].

The most frequent causes of mildly enhancing lymph nodes are lymphoma, sarcoidosis, or lung cancer metastases. Notably, lymphomas usually grow and expand along the existing structures, while lung cancer metastases may have a mass effect or an infiltrative growth [85,86,87].

Hyper-enhancing nodes are suggestive for metastases from hypervascular cancers (i.e., melanoma, renal cell carcinoma) or for Castleman disease [78,88].

Hypo-attenuating lymph nodes usually correspond to cystic or necrotic degeneration (e.g., metastases from lung or testicular cancers) [15]. The presence of ring enhancement surrounding the central necrotic core can correspond to infectious diseases, such as mycobacterial or fungal infection. Anamnestic information (such as travels in endemic areas) together with clinical symptoms and pulmonary findings may orient the diagnosis to pulmonary and mediastinal infections [89,90].

Calcified lymph nodes can be observed in several conditions, mostly granulomatous diseases. Healed or remitted tuberculosis may present completely calcified lymph nodes, usually asymmetrical. In sarcoidosis or silicosis, the mediastinal enlarged lymph nodes are symmetrical and bilateral, with variable pattern of calcification (e.g., peripheral or eggshell, diffuse, central, or hazy). The clinical and anamnestic data, together with pulmonary findings, are helpful to achieve the correct diagnosis [75,78,86,91,92]. *Mediastinal lipomatosis* is the excess adipose tissue mostly seen in obese patients or after steroid therapy. On CXR it may occasionally appear as a massive lesion; however, chest CT confirms the normal finding of diffuse, unencapsulated tissue with homogeneous fat attenuation surrounding anatomic structures in all mediastinal compartments [93].

## 3. Differential Diagnosis of Pleural Lesions

Pleural disease is first suspected on CXR only in advanced cases because of the presence of indirect signs (i.e., pleural effusion or pneumothorax); however, CT and MRI are the modalities of choice for further characterization [94,95,96]. On CT, the first step is to determine whether the lesion comes from the pulmonary parenchyma or the pleura: the presence of an acute angle between the lesion and the thoracic wall means a pulmonary origin, while an obtuse angle suggests a pleural disease [97]. Pleural lesions, unlike the extrapleural ones, usually do not cause the erosion of the ribs or the outward displacement of the extrapleural fat [97]. Pleural lesions can be grouped into tumor (benign and malignant) and tumorlike conditions [98]. Malignant neoplasms are more common than benign neoplasms. *Pleural thickenings* can be focal (>5 mm) or diffuse (>25% of the chest wall if bilateral and >50% if unilateral) [99]. Imaging features suggesting pleural malignancy (primary or secondary) are nodular (>1 cm) pleural thickening, with circumferential involvement (mostly for mediastinal pleural), enhancement after contrast media administration, and the presence of unilateral pleural effusion [100,101]. CT is the gold standard for the evaluation of pleural disease; a late-phase acquisition (70 s after the contrast media administration) further improves the accuracy [102].

A potential pitfall in the identification of focal, non-calcified, pleural thickening in the posterior–basal location at supine scans is the focal accumulation of lymphatic fluid within pleural layers because of gravity; in these cases, an additional prone acquisition could rule out diagnostic doubt [103,104].

### 3.1. Pleural Neoplasms

Most of the features mentioned above are typical for primary pleural malignant neoplasm, the most common of which is *pleural mesothelioma* (PM) [105]. It demonstrates a very aggressive behavior, invading the mediastinum, chest wall, and diaphragm and often showing lung nodules and carcinomatous lymphangitis [106,107]. These patients are usually in their sixth or seventh decade of life, they have a history of exposure to asbestos fibers (average latency of 35–40 years), and they can show other signs of asbestosis, such as benign pleural plaques (average latency of 20 years) [108,109].

Although PM is the most common primary malignant pleural tumor, the first cause overall of pleural effusion and tumor nodules is *metastatic pleural disease*. Differentiation from PM is difficult: unilateral involvement and volume loss of affected the hemithorax favors PM [98,108]. Common cancers that metastasize to the pleural space are breast, lung, lymphoma, ovary, and gastrointestinal primary carcinomas [91]. Therefore, when CT images suggest malignant pleural disease, the presence of a primary tumor must be ruled out [110].

The *solitary fibrous tumor* is a benign, relatively uncommon pleural neoplasm with a peak incidence in patients >50 years of age. It is often misinterpreted by inexpert radiologists since it typically appears like a solitary, lobulated soft-tissue mass usually involving the inferior hemithorax with areas of necrosis, hemorrhage, calcifications (up to 26%), cystic changes, and with heterogeneous enhancement [100,111]. In 20% of cases, they can be malignant; the findings suggesting malignancy are the presence of calcification, effusion, atelectasis, mediastinal shift, and chest wall invasion [112].

Other primary pleural malignant tumors to be considered are *pleural sarcomas* and *primary lymphomas*; however, they are very rare.

### 3.2. Tumorlike Pleural Lesions

As mentioned before, *pleural plaques* can be calcified or not and are the most common manifestation of asbestos exposure occurring with a latency of 20–30 years. However, there is no risk of malignant degeneration, but these patients may have an increased risk of PM and lung carcinoma because of the exposition to toxic agents [110,113]. These lesions are a quite common incidental finding since they are asymptomatic and usually are located on the posterolateral aspect of the lower ribs, parietal pleura, and the diaphragm dome while the visceral pleura, costophrenic angles, and lung apices are characteristically spared. In the case of non-calcified plaques, the differential diagnosis with pleural carcinomatosis may be challenging. In such cases, it has been demonstrated that iodine maps, obtained from dual-energy CT (DECT) acquisition, are helpful to differentiate non-calcified benign pleural lesions from pleural carcinomatosis with a higher sensitivity and specificity than conventional CT [114].

*Thoracic splenosis* should be considered in case of pleural, highly enhancing nodules (multiple or solitary) in patients with a history of splenic or diaphragmatic trauma, or surgery. It is a tumorlike condition caused by the autotransplantation of splenic tissue into the pleural cavity (latency of <10 years). Usually, the lesion has the same imaging features as the spleen (if still present); however, there can be doubtful cases, mostly because the nodules increase in size. In such cases, the gold standard for diagnosis is scintigraphy with ^99m^Tc heat-damaged tagged erythrocytes [115].

A similar condition, but very rare, is the *thoracic endometriosis*, since the pleura is the most common extra-abdominal location for endometrial tissue. In this case, the ectopic implant of endometrial tissue in the pleural cavity may cause back pain, pneumothorax, or recurrent hemothorax, with the onset of menses in childbearing women with a history of endometriosis [115]. Furthermore, in this case, pleural highly enhancing nodules (multiple or solitary) are present; they characteristically reveal cyclical changes in temporal relation with menses and can present the distinctive posterosuperior location of diaphragmatic lesions [116].

Another rare but characteristic diagnosis is *Erdheim–Chester disease* (ECD), which is a multisystemic disorder classified as a non-Langerhans cell histiocytosis [117]. It should be suspected in the case of symmetric circumferential pleural thickening or effusion, smooth interlobular septal thickening, and pericardial thickening. Additional findings are the characteristic perirenal soft-tissue encasement (“hairy kidney sign”) and symmetric skeletal abnormalities as long bone sclerosis often involving the distal femoral meta-diaphyses [98,117]. The extraosseous disease occurs in half of the patients, and the central nervous system, lungs, heart, and retroperitoneum may also be affected; chest involvement has been reported in less than half of patients affected by this disease. Long bone radiographs and radionuclide bone scintigraphy should be performed to confirm this rare diagnosis [98,117].

The IgG4-related disease is a rare immune condition characterized by increased serum levels of IgG4 with organ infiltration by IgG4-positive plasma cells or lymph plasmacytes. Even though the more frequent manifestations include the abdominal district (e.g., autoimmune pancreatitis, nephritis, sclerosing cholangitis, lymphadenopathy, retroperitoneal fibrosis, and sclerosing mesenteritis), up to 13% of patients present pleuropulmonary involvement [118,119]. The pleural manifestation is mainly with a focal thickening of the parietal or visceral pleura, with or without parenchymal involvement [120]. On the other hand, pulmonary involvement has a variable presentation (from solid nodules or mass-like lesions to alveolar and interstitial involvement) [121].

The imaging appearances of previous treatments (until the 1950s) of tuberculosis may have an historical interest in very elderly patients [122]. A common theory behind these treatments was that the collapsed parenchyma would accelerate the healing. This was achieved by placing inert materials such as acrylic balls, rubber sheets, or oils in surgical cavities (*plombage*), or by direct intra- or extra-pleural injection of paraffin oil (*oleothorax*), to treat bronchopleural fistulas, empyema, or pneumothorax [123,124]. These materials have various radiographic appearance and are usually detected as pleural or subpleural masses [123,124].

## 4. Differential Diagnosis of the Parenchymal Disease

CXR is usually the first examination performed to rule out pulmonary diseases because of its high availability and sensitivity. However, even with the efforts for improvements, it lacks specificity [2,125]. The evaluation of parenchymal lesions mainly relies on CT; with recent technological improvements, it is possible to perform low-dose CT (LDCT) with image quality comparable to the conventional CT [2,126,127]. This is valuable mostly for patients that need several follow-up CT (e.g., chronic lung diseases, evaluation of the response to treatments) and has opened the possibility to CT lung cancer screening [127,128,129,130,131]. Other advanced imaging modalities (i.e., chest-MRI or FDG–PET) are more useful for lung cancer staging than for diagnostic purposes, also allowing for the provision of a more precise target for potential biopsies [132,133,134,135].

### 4.1. Focal Lung Involvement

According to the Fleischner Society, a “lung nodule” is a rounded opacity, well or poorly defined, measuring up to 3 cm in diameter; if the lesion is >3 cm it is defined as a “mass” and should be considered indicative of lung cancer until histologically otherwise proven [79,136]. With the terrific increase in the number of CT performed yearly, the incidental detection of solitary pulmonary nodules (SPNs) has consequently increased, with an overall reported incidence of 8–51% [137,138]. The differential diagnosis for SPN is extremely broad: given the poor prognosis of lung cancer, the first step is to assess the likelihood of early lung cancer. The Fleischner Society proposed a model for the management of incidentally detected SPN in patients >35 years old, not immunocompromised, and not with known primary cancers; this model stratifies the risk based on the SPN size and presence of risk factors (i.e., smoking, emphysema and fibrosis, family history of lung cancer) [139].

Size and growth positively correlate with the likelihood of malignancy; however, just as malignancy is not excluded in small SPN, growth or big nodules are not unequivocally signs of malignancy [140]. The evaluation of the SPN considers clinical factors and morphology, and the radiologist must be aware that imaging features of benign and malignant nodules may overlap [141]. PET/CT with 18F-FDG can be an additional tool, since small nodules (<8 mm), adenocarcinoma precursors or with lepidic growth, and carcinoids can show low or no uptake [142]. Therefore, the second step is to carefully evaluate the density (solid or subsolid), morphology (shape, margins), composition (fatty, cavitations, and calcifications), and additional findings. Table 3 summarizes some general features to take into consideration in this process. Some more detailed considerations for the main morphological features to evaluate at CT imaging are reported below.

Typical features suggestive for malignancy (i.e., primary lung cancer) are a solid nodule >1 cm with spiculated margins, lobulated borders, irregular shape, showing a significant enhancement (20–60 HU) after contrast media injection, or volume doubling time (VDT) in the follow-up CT scans of 30–400 days; possible additional findings as pleural tags or satellite nodules are also considered [143,144]. Regarding the VDT, the equation used for calculation assumes a constant cellular division rate with exponential volume increase [145]. The VDT for solid nodules is well established, being 30–400 days for the majority of them. Conversely, subsolid nodules have a more indolent growth with a VDT of 3–5 years [146,147]. This results in longer follow-up intervals and periods for the subsolid nodules [139,148].

If the nodules (or masses) are multiple, with soft-tissue attenuation, varying in size, with basal predominance (due to blood flow), then metastatic disease is the first hypothesis to be investigated [149]. Primary tumors that commonly metastasize to the lungs are breast, colon–rectum, kidneys, head and neck, and thyroid cancers [149].

In the last years, the American College of Radiology (ACR) has coordinated the development of the Lung CT Screening Reporting & Data System (Lung-RADS), recently updated with version 2022 [150]. The algorithm is based on the imaging characteristics of the nodules detected on LDCT in the screening setting and provides a five-point score with increasing probability of malignancy. For each category, the Lung-RADS system also provides the recommendation for the management of observation, from follow-up with LDCT to the integrated diagnostic workup in case of suspicious nodules [151]. The CT features included in the Lung-RADS system are discussed in the next sections.

#### 4.1.1. Density

**Figure 3:**
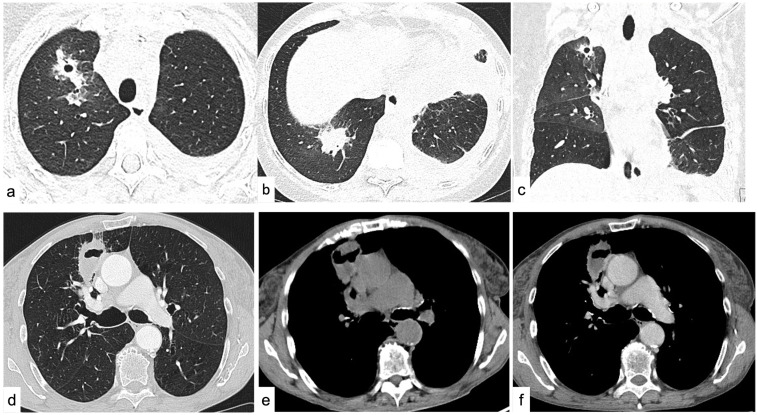
Pulmonary aspergillosis vs. cavitated metastases by squamous cell carcinoma of the tongue. Different cavitated lesions showing spiculated margins: pulmonary aspergillosis. Axial (**a**,**b**) and coronal (**c**) view of multiple solid cavitated nodules, with spiculated margins and peripheral ground glass (halo sign) in a 43-year-old neutropenic patient with follicular non-Hodgkin lymphoma (NHL), presenting with fever and cough. Cavitated metastases by squamous cell carcinoma of the tongue. Axial CT scans before (**d**,**e**) and after iodinated contrast medium administration (**f**) showing cavitated nodule in the paramediastinal site in a 67-year-old female patient with history of squamous cell carcinoma of the tongue.

The first step is the assessment of the attenuation of the SPN and to define whether it is solid or subsolid. If the nodule obscures the underlying structures (bronchial and vascular), it is defined as “solid” [79]. A “*subsolid nodule*” contains a proportion of ground-glass opacity (GGO), where the underlying structures are still visible through the higher density parenchyma; in this case, it is a “*part-solid nodule*”, otherwise, if the whole nodule is composed of GGO, it is a “*pure GGO nodule*” [79]. The Early Lung Cancer Action Project (ELCAP) highlighted that subsolid nodules have a significantly higher risk of malignancy compared to solid nodules, respectively, 34% versus 7%, and should usually undergo close follow-up (3 months) [152,153]. In part-solid nodules, the GGO component may assume the so-called *halo sign*, where the GGO circumscribes the solid component of the nodule. In malignant nodules, the halo sign is caused by local tumor spread (i.e., lepidic growth pattern) where tumor cells proliferate along the surface of intact alveolar walls without stromal or vascular invasion [154,155]. These lesions are recognized as *minimally invasive adenocarcinoma* (MIA) and *lepidic predominant adenocarcinoma* (LPA) by the new World Health Organization (WHO) classification [156]. However, the “halo sign” can be related also to benign conditions such as eosinophilic pneumonia, organizing pneumonia, tuberculosis, cytomegalovirus, herpes simplex virus, and aspergillus infection (this mostly for patients affected by neutropenia). However, in these cases, the nodules usually disappear at the follow-up [157,158]. Conversely, a malignant part-solid nodule persists at the follow-up and characteristically changes in morphology, rather than size, with an increase in the solid component over the GGO (Figure 3) [139,159,160,161].

Evaluation of the growth at the follow-up can be challenging, mostly for part-nodules, because of their slow growth; therefore, evaluation with computer-aided volumetry is recommended [139]. The complement of the “halo sign” is the “*reversed-halo sign*” where the GGO is surrounded by a ring of consolidation; this is related to cryptogenic organizing pneumonia or to lung cancer nodules after radiofrequency ablation [162].

#### 4.1.2. Shape

The typical SPN has a round or oval shape [163]. It is important to evaluate the morphology of perifissural nodules since they commonly represent intrapulmonary lymph nodes (ILN) [164]. ILN are small (<10 mm), triangular or polygonal, elongated in shape, and characteristically lie within 15 mm of a pleural surface [140,164,165]. ILN may present the same doubling time (DBT) as malignant nodules, and this is not considered a sign of malignancy; conversely, the presence of spiculated margins or crossing the fissure requires further workup [140,166,167].

Another characteristic benign entity is the *rounded atelectasis*, which can be found in relation to previous pleural effusion or history of asbestos exposition with pleural thickening or pleuritis [168]. On CT it demonstrates a characteristically rounded, mass-like appearance with a predilection for the lower pulmonary lobes, and it is strictly related to the pleural surface [168,169]. Rounded atelectasis may have the classical “comet tail sign” due to the pulling of the bronchovascular bundles close to the lesion and, less commonly, the presence of some linear bands raising from the lesion (“*crow feet sign*”) [170,171]. It is crucial to recognize this entity, since, as it represents a collapsed lung, it commonly demonstrates a typical parenchymal enhancement and could be misinterpreted as malignant [168,169].

#### 4.1.3. Margins

The margins can be defined as *smooth*, which usually are associated with benignity, or with *lobulation* or *spiculation*, more commonly related to the presence of malignancy. However, this is not a “golden” rule since it is reported that about 21–33% of malignant SPNs demonstrate smooth margins, and lobulated margins can be seen in benign hamartomas [142,172,173]. Conversely, spiculations are highly predictive of malignancy (positive predictive value of 90%) and are a sign of invasiveness [173,174,175]. While measuring a spiculated SPN, the spiculation should be kept out of the measurement to improve the reproducibility [139]. It should be noted that also some benign conditions demonstrate spiculated margins, such as infection tuberculomas, inflammatory pseudotumors, focal atelectasis, and fibrosis; however, in such cases, the malignant hypothesis should be always ruled out [175].

An additional finding to be considered is the presence of pleural retraction (pleural tag). This is more common in malignant SPNs; it is a sign of invasiveness but it is rare in metastatic disease or carcinoid [79,174,176,177].

#### 4.1.4. Fat Attenuation

As previously mentioned for mediastinal lesions, fat content can be easily assessed on CT with a soft-tissue window; the attenuation values range from −40 to −120 HU [178]. Intralesional fat is highly suggestive of *hamartoma*; these benign lesions usually show dimensional stability over time, well-defined lobular or notched borders, and calcification with a “popcorn” shape (see below) [144,172]. However, 50% of hamartomas do not show intralesional fat on CT; MRI with chemical shift sequences is more effective than CT for the detection of intralesional fat that can be associated to other imaging findings of hamartoma [179]. The additional findings of hamartoma should be always detected since metastases from liposarcoma and renal cell carcinoma may present as SPN with fat content.

#### 4.1.5. Calcifications

The presence of calcification in an SPN can be easily evaluated with the bone window and it is widely considered a sign of benignity [180]. However, the different morphology of the calcification may suggest malignancy or benignity. Specifically, the presence of diffuse, central, lamellated calcification of SPNs suggests the benign etiology [181]. These patterns could be associated with prior infections such as histoplasmosis or tuberculosis, and they are usually multiple, a few millimeters in diameter, and represent granulomas [181].

As previously mentioned, the “popcorn-like” calcifications are considered characteristic of pulmonary *hamartoma* when found in combination with intralesional fat [172,178]. Eccentric, dystrophic, or punctate calcifications are indeterminate, sometimes benign, and related to granulomatous disease; they also occur in malignant lesions such as carcinoid tumors, lung cancer, and metastatic disease (osteosarcoma, chondrosarcoma, and mucinous adenocarcinoma); they are not a helpful discriminator between benign and malignant nodules (Figure 2) [142,182,183].

#### 4.1.6. Cavitations and Cysts

A cavity is defined as a gas-filled space within a pulmonary consolidation, mass, or nodule [79]. It can be associated with either a neoplastic (primary lung cancer, metastasis) or an infectious disease (including bacterial, mycobacterial, and fungal infections) as well as inflammatory disease (e.g., vasculitis, pulmonary Langerhans cell histiocytosis) [184]. Smooth and thin walls (usually <5 mm) are usually associated with benign conditions; on the other hand, thick (>15 mm), irregular, and nodular walls, are associated with malignancy. However, there is great overlap between benign and malignant, since lung cancer can occur as cysts with thin walls or lung abscesses can have a thick wall [185]. The association of other imaging features can help in the differential diagnosis: ground glass, consolidation, bronchial wall thickening, and satellite nodules are indicative of a benign nature of the cavitary SPN. The presence of an inner fluid level, rim contrast enhancement, and ancillary findings of pulmonary infection (e.g., consolidation, tree-in-bud opacities, pleural fluid) may be suggestive of a *lung abscess* (Figure 1) [186]. Cavitations could also occur after chemo- and radiotherapy, or antiangiogenic agents, and they are thought to be secondary to tumor necrosis or a valve effect on an adjacent bronchus [184].

**Figure 4:**
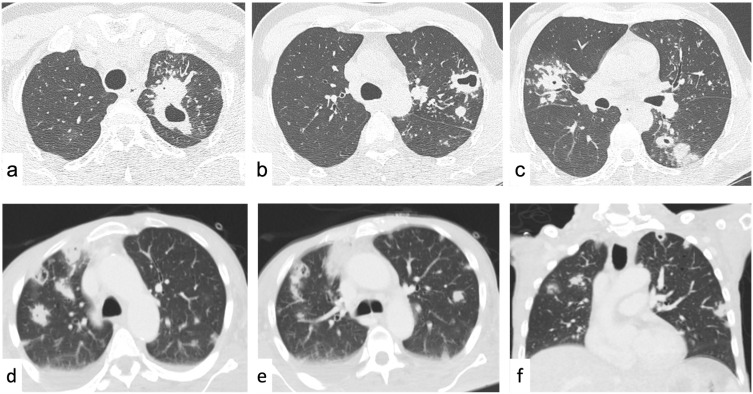
Excavated nodules vs. septic emboli. Axial CT scans (lung window) in a 48-year-old male patient with asthenia, fever, and productive cough for several days (**a**–**c**). It shows multiple solid nodules and micronodules with centrilobular distribution, with a “tree-in-bud” pattern, larger nodules, and some excavated lesions at the upper lobes and apices (tuberculosis). Axial CT scans (**d**,**e**) with coronal reconstruction (**f**) in a 54-year-old male patient presenting with fever and laboratory evidence of septic shock caused by the presence of infective endocarditis from Staphilococcus Aureus. Solid nodules, some with cavitation and peripheral ground glass, joined with the clinical data suggested the diagnosis of septic emboli.

If the cavitated lesions are multiple and show similar morphology one each other, the differential diagnosis is among an infectious (TBC or fungal infections), inflammatory (vasculitis), or metastatic disease (squamous cell lung cancer, head and neck, gastrointestinal adenocarcinomas, sarcomas, breast cancer); all of them can show the “feeding vessel sign” (a pulmonary artery branch leading to the SPN) (Figure 4) [149].

*Metastases* usually demonstrate a diffuse distribution, while septic emboli usually are peripheral, multiple, round or wedge-shaped, with lower lobe predominance, and may not show contrast enhancement [187]. The suspicion of septic emboli can be supported by the presence of predisposing conditions of infective foci spread (infective endocarditis, infected deep venous thrombosis, periodontal disease) or the presence of some medical devices favoring infection (catheters, venous lines, central venous catheters, pacemaker wires) (Figure 1, Figure 3 and Figure 4) [188,189].

*Fungal infections* (*Aspergillus* spp. or *Cryptococcus* spp.) can be suspected in an immunocompromised patient, with acute onset of infective state with nodules possibly showing the “halo sign” (due to surrounding hemorrhage) (Figure 3) [190]. *Granulomatosis with polyangiitis* (GPA) is another possible condition to consider in a non-oncological patient, presenting with multiple lung nodules with cavitation, random distribution, and different size and morphology. It is a necrotizing non-caseating granulomatous vasculitis of small to medium-sized vessels affecting lungs, kidneys, and airways [191,192]. Characteristically, the nodules of GPA tend to “migrate” over time, modifying their location and size; this finding paired with the presence of lesions of the upper respiratory tract and c-ANCA positivity is strongly suggestive of GPA [192,193].

The best way to assess the diagnosis in cavitated nodules is a short-term follow-up since infectious (bacterial) or inflammatory cavitation often presents with rapid changes and correlation with clinical data and blood cultures [194].

Other air-containing signs are the *broncogram*, *intranodular bubble-like lucencies*, or *cystic airspaces*. The *air bronchogram* in the setting of lung consolidation has to be considered benign; however, in the context of an SPN, it is more commonly associated with malignancy [79,195,196]. In addition, the presence of intranodular, bubble-like lucencies or cystic airspaces is common in neoplastic nodules [79,197,198,199]. Specifically, the progressive wall thickening or the presence of a nodule in- or outside a cystic airspace should raise the suspicion of malignancy [200]. These findings have been recognized as a feature of early malignant disease; thus, a short-time follow-up or a specific diagnostic workup is recommended [201]. These concepts have been included in the latest version of Lung-RADS with the definition of “Atypical Pulmonary Cyst” [141,151].

### 4.2. Diffuse Lung Disease

The first step in diffuse lung disease is to recognize the predominant pattern and its distribution along the pulmonary parenchyma on a high-resolution CT (HRCT). Four main patterns are described: reticular pattern, nodular pattern, and increased and decreased lung attenuation [202].

#### 4.2.1. Reticular Pattern

Reticular opacities at HRCT scans indicate the presence of diffuse lung infiltration; three principal patterns may be seen: interlobular septal thickening (smooth and nodular), honeycombing, and irregular reticulation [1,79].

##### Interlobular Septal Thickening

Smooth interlobular septal thickening—In patients with acute dyspnea and chronic heart failure, it is commonly related to *pulmonary edema*. It is characteristically bilateral and symmetric with predominance in the lower lobes, and it is related to the fluids overload into the septal lymphatic vessels [1]. Conversely, in an oncological patient (mostly breast, lung, or gastrointestinal tract cancers), this finding is suspicious for early *pulmonary lymphangitis carcinomatosis* (PLC) [203]. In this case, as for other regions, the lymphatic vessels outflow is impaired by the metastatic infiltration and will progress in nodular septal thickening [203,204,205,206]. Associated findings to PLC are the presence of hilar and mediastinal lymphadenopathy, “peribronchial cuffing”, and pleural effusion [207,208]. Similarly, in lymphoid interstitial pneumonia (LIP), a rare, benign lymphoproliferative disorder, lymphocytic proliferation leads to smooth interlobular septal thickening; however, this is an ancillary finding since the disease is mainly associated with thin-walled cysts (see below) [209]. Two rare conditions that can be associated with smooth septal thickening are Erdheim–Chester disease (ECD) and the Niemann–Pick disease (type B); these conditions usually do not have a primary lung involvement and the diagnosis can be suspected on other extrapulmonary findings. Specifically, ECD may also present pleural thickening or effusion, “hairy kidneys”, and osteosclerosis of the long bones [210]. Conversely, Niemann-Pick type B disease is associated with hepatosplenomegaly with or without calcifications, calcified lung nodules, and early development of atherosclerosis disease [211].

Nodular interlobular septal thickening—It is a sign of cellular distribution within the septal lymphatics; therefore, the first differential diagnosis to be ruled out is PLC [1,79]. However, when symmetrical, bilateral mediastinal lymphadenopathy (often calcific) is present in a non-oncological patient, <40 years of age, *sarcoidosis* should be considered. Nodules usually have a perilymphatic distribution involving subpleural, peribronchovascular (mostly in perihilar regions), and centrilobular interstitium and tend to be distributed in a patchy fashion. Additionally, erythema nodosum can be present [212,213]. *Pneumoconioses* (silicosis and coal workers’ pneumoconiosis) have a quite similar appearance to sarcoidosis and can be indistinguishable at CT scans. However, pneumoconioses usually are associated to a sign of fibrosis because of exposure to inorganic dust, and a clinical history of exposure is necessary for the diagnosis [214,215]. A rare pathology associated with this pattern is *amyloidosis*; this is a complex and variegate pathology characterized by extracellular accumulation of amyloid. Since the lungs are not one of the primary organs involved, it is better to first evaluate the presence of suspicious findings in other districts (kidneys, heart, nervous system, and liver) [216].

##### Honeycombing

Honeycombing is defined as clustered cystic airspaces, typically of comparable diameters (3–10 mm) with well-defined walls (1–3 mm) and subpleural distribution [79]. Occasionally, cystic airspaces can be as large as 25 mm (macrocystic honey combing). Honeycombing is the most specific sign of fibrosis, and can be associated with heterogeneous subpleural reticular opacities, traction bronchiectasis, and a lower lobe predominance to give the “*usual interstitial pneumonia (UIP) pattern*” [217]. Conversely, in absence of honeycombing, the presence of reticular pattern with subpleural basal predominance defines the “*probable UIP pattern*”. Other features, such as mild ground-glass opacities (GGOs) or distortion, or subtle reticulation with subpleural and basal predominance, define the “*indeterminate pattern for UIP*”. Finally, the presence of other findings such as cysts, mosaic attenuation, predominant GGO, nodules and consolidations, with peribronchovascular or perilymphatic distribution at the upper or mid lungs, suggests an “*alternative diagnosis to UIP*” [217].

The UIP pattern includes in the differential diagnosis idiopathic pulmonary fibrosis (IPF), connective tissue diseases (CTD), asbestosis, drug-induced lung disease (DILD), and chronic hypersensitivity pneumonitis (HP) [1].

IPF is the term for the clinical syndrome associated with the CT pattern of UIP, and it is associated with smoking [218,219]. The presence of a “probable UIP pattern” or “indeterminate pattern for UIP” does not rule out the diagnosis of the UIP/IPF, since it can be detected at pathology in nearly 30% of cases for each of the two CT patterns [220]. However, if a patient with a UIP pattern has a disease or exposure that is known to be associated with this pattern (e.g., collagen disease and asbestos exposure), by definition, the diagnosis cannot be IPF [1,218]. Therefore, in the presence of a UIP pattern at the HRCT scan, always think of an IPF and exclude all the other known causes for a UIP pattern [1,218]. Since IPF is one of the interstitial pneumonias (IP) with the worst prognosis, a lung biopsy should be performed for diagnosis of certainty in case of probable or indeterminate CT patterns of UIP [218,220]. Different density scores have been developed to assess lung involvement in IPF, but quantitative scores demonstrated the best correlation with lung function [221]. Since these patients usually have a history of smoking, it is important to correctly differentiate honeycombing from paraseptal emphysema where the cysts present thin walls and are distributed in a single layer (Table 4) [222].

Mild honeycombing (microcystic) could be present in advanced stages of *nonspecific interstitial pneumonia* (NSIP); this is in combination with patchy GGO, irregular linear or reticular opacities, and scattered micronodules [218]. Unlike IPF, NSIP findings have homogeneous distribution (no obvious gradient) with typical subpleural sparing and usually demonstrate a good response to corticosteroid therapy, and exposure to cigarette smoking does not seem to be related. As UIP, NSIP could be related to CTD, HP, and DILD. NSIP is the most common pattern seen in patients with CTD; specifically, it is most typical of scleroderma, polymyositis, dermatomyositis, and mixed CTD. The UIP pattern is more common in rheumatoid arthritis.

##### Irregular Reticulation

The presence of irregular reticulation is indicative but nonspecific of fibrosis, and it may be seen in association with other reticular findings that could help in diagnosis. As previously mentioned, when associated with honeycombing and traction bronchiectasis (UIP pattern), it can be assumed to reflect fibrosis. In this case, it is the presence of honeycombing that is most important for the differential diagnosis.

When irregular reticulation is associated with GGO and traction bronchiectasis is absent, consider an infiltrative or inflammatory disease as the most likely (NSIP pattern). In this setting, consider *radiation-induced lung diseases* (RILD) in the acute phase for oncologic patients who underwent chest radiation therapy (latency <6 months) who demonstrate fatigue and respiratory symptoms [223,224,225,226]. In the acute phase of RILD, GGO can be associated with consolidations in the region of treatment and may completely resolve or evolve to volume loss, and reticular septal thickening with traction bronchiectasis in the late phase [227]. Differential diagnosis is often required to exclude infections (which have characteristic clinical data and abrupt onset), lymphangitis carcinomatosis (which usually evolves in worse symptoms and radiological manifestations), and malignancy recurrence (for which usually FDG—PET could be diriment) [227].

CTD and DILD are also in the differential diagnosis for irregular reticulation associated with GGO. Pleural and pericardial thickening or effusion may coexist and may be useful for diagnosis. In scleroderma, associated findings such as pulmonary hypertension and esophageal dilation (up to 80% of cases) may be present. DILD (e.g., by chemotherapy or amiodarone) is a challenging diagnosis; it may be associated with several possible appearances on HRCT. The most common patterns of lung injury associated with DILD include NSIP and UIP patterns, pulmonary edema, pulmonary hemorrhage, diffuse alveolar damage (DAD), organizing pneumonia (OP), and eosinophilic pneumonia [228]. These CT patterns are associated with drug toxicities, but there are no HRCT findings that specifically suggest drug toxicity. The exception is the presence of hyperdense consolidations that are highly suggestive for amiodarone and its metabolites accumulation into macrophages [229]. A high degree of suspicion and correlation with medication history is necessary to make a confident diagnosis.

When associated with air trapping in a post-expiratory scan, irregular reticulation can be related to chronic HP, chronic sarcoidosis, or CTD-ILD [1].

The statements above are the simplification of a complex topic regarding the IP, where often the differential diagnosis between UIP and NSIP patterns is not plain; for these reasons, structured reporting and a multidisciplinary evaluation are recommended [230,231,232].

#### 4.2.2. Nodular Pattern

The nodular pattern is defined by the presence of multiple roundish pulmonary opacities ranging in diameter from 2 to 10 mm as the predominant finding [79]. The HRCT evaluation is based on morphology, density, and distribution in the craniocaudal direction, and the relation to the secondary pulmonary lobule (perilymphatic, centrilobular, or random) of the nodules [1,79].

##### Perilymphatic Nodules

Nodules with a perilymphatic pattern are characteristic of diseases involving the pulmonary lymphatics [1,233]. It grossly replicates the same pathological scheme of the nodular interlobular septal thickening mentioned above; it can be in association with granulomatous disease (sarcoidosis, pneumoconiosis), amyloidosis, or neoplastic diseases (adenocarcinoma, lymphoma, PLC) [1,233]. The differential can be made through the investigation of the patient’s history and the presence of an ancillary sign, as the “*galaxy sign*” for sarcoidosis where the nodules coalesce in a large parenchymal nodule [79,234].

##### Centrilobular Nodules

Centrilobular nodules occur in diseases that primarily involve structures at the center of the secondary pulmonary lobule (bronchiole, artery, or peribronchiolar lymphatics) [1,79,235]. Characteristically, the nodules demonstrate regular spacing among each other and sparing of subpleural interstitium [79,233,235]. This pattern is more frequently related to the peripheral airway disease, and when it is in association with other smoked-related features such as emphysema, predominantly in the upper lobes, the diagnosis of *respiratory bronchiolitis—interstitial lung disease* (RB-ILD) can be placed confidently [218,219,236]. Nevertheless, if centrilobular nodules are observed in a non-smoker patient with a history of inhalation of specific antigens (usually organic such as fungi, bacteria, protozoa, or animal proteins), suffering from episodes of acute illness with dyspnea and coughing, it could be suggestive of *hypersensitivity pneumonitis* (HP) [237]. In acute onset, it is possible to see the combination of patchy ground-glass opacities, normal regions, and low-attenuated areas (expression of air trapping), often described as a “three-density pattern” (or “headcheese sign”). Bronchiolar wall thickening and mediastinal lymph node enlargement are frequently associated [238]. Centrilobular, ill-defined nodules together with with patchy GGO and tree-in-bud are usually related to the *endobronchial spread of bronchiolitis* (bacterial, viral, or fungal). The association with acute symptomatology (e.g., fever and cough) and laboratory evidence of infection are useful to confirm the CT suspicion [233,235,239].

Another structure within the central lobe is the central arteriole, which can be involved in vasculitides. Specifically, microscopic polyangiitis, eosinophilic granulomatosis with polyangiitis, and giant cell arteritis can present pulmonary involvement with centrilobular nodules [240,241].

##### Random Nodules

Random nodules have no specific pattern of distribution to the lung structures or the pulmonary lobule [79,233]. Several conditions with hematogenous spread are associated with random nodules. The most common conditions depicted by this pattern are *hematogenous metastases*: these nodules are usually of soft-tissue attenuation and may show a basilar predominance in size and number [1]. Other conditions can be septic emboli and miliary tuberculosis [233]. Similar findings can be related to *post-primary tuberculosis* (TBC) in immunocompromised patients, but it is usually associated to lymphadenopathy, pulmonary consolidation, and pleural effusion [242]. Further laboratory tests are needed for the diagnosis of TBC [242].

#### 4.2.3. Increased Lung Attenuation

Increased lung attenuation includes two categories of findings: ground-glass opacities and consolidations.

##### Ground-Glass Opacities

Ground-glass opacities (GGOs) have been defined above; they do not represent a specific HRCT finding, as may represent either alveolar disease or interstitial disease [79]. Association with acute (<6 weeks) or chronic (>6 weeks) symptoms could be helpful for the differential diagnosis [243,244].

Patients with acute respiratory symptoms and HRCT depicting patchy and peripheral ground-glass opacities (GGOs), in combination with smooth septal thickening and airspace consolidation, are likely to be affected by pneumonia, especially COVID-19. This new entity has partially changed the diagnostic workflow since CT findings can be supportive of the diagnosis of COVID-19 in patients with a negative nasopharyngeal swab, thanks to the high sensitivity and good interreader agreement of CT [245,246,247,248]. Even if the role of CXR is still debated, it is unequivocally useful in bedridden patients for evaluation of response to treatments, especially in patients treated with extracorporeal membrane oxygenation (ECMO) in an intensive care unit (ICU) [249,250,251,252]. Some authors also proposed MRI as a more sensitive imaging modality that does not require radiation exposure in the follow-up of COVID-19 patients [253]. Several scores and CT signs were developed in an attempt to predict prognosis, hospitalization, and response to treatments; however, they are usually time-consuming and poorly applicable in daily clinical practice [254,255,256,257,258,259,260,261,262,263]. In this setting, artificial intelligence is showing promising results for reducing the workload, especially in the oncological setting [264,265,266,267,268,269,270]. Several studies have suggested an increased incidence of pulmonary embolism in COVID-19 patients, with associated higher mortality [271,272,273,274,275,276,277,278]. In the pandemic setting, the significant increase of requests for CT and CT pulmonary angiography examinations, often repeated in hospitalized patients, have raised concerns about the radiation protection and iodine load. The use of CT protocols optimized with iterative reconstructions and artificial intelligence, and dual-energy CT, are helpful in this condition and in particular in younger patients [279,280,281].

In patients affected by chronic heart failure, with acute symptoms, the presence of *acute pulmonary edema* should be investigated. As previously mentioned, it is characterized by diffuse and bilateral GGO and interlobular septal thickening; the presence of heart enlargement can be helpful in cases of unknown patient history or when the underlying condition is unknown [282].

In patients with hemoptysis, anemia, and hypoxemic respiratory failure, *diffuse alveolar hemorrhage* (DAH) is the most probable cause of patchy lobular GGO. When HRCT and clinical features are suggestive of DAH, progressively hemorrhagic bronchoalveolar lavage (BAL) found in serial samples is diagnostic of DAH [135,283,284].

In oncological and immunocompromised patients showing GGO associated with smooth septal thickening with a slow onset of dry cough and dyspnea, it is important to take into consideration opportunistic and atypical infection, such as *Pneumocystis Jiroveci Pneumonia* (PJP) [285]. Imaging findings for this pathology are not specific at onset; then, paired with rapid respiratory deterioration, diffuse areas of crazy-paving pattern and patchy airspace consolidations occur in association with the presence of lung cysts [285,286].

Some diffuse lung diseases are associated with GGO, both in the acute and the chronic setting. These are *eosinophilic pneumonia* and *hypersensitivity pneumonitis* (HP). *Acute eosinophilic pneumonia* (AEP) is a quite rare entity that should be suspected in patients with bilateral areas of GGO associated with thin nodular septal thickening. These patients demonstrate a serologic increase in eosinophils and hypoxemia after an acute febrile illness. Since peripheral blood eosinophils are usually normal in the acute setting at presentation, this serological finding during the course of disease should raise suspicion of AEP. The definitive diagnosis can be confirmed by the prompt response to corticosteroids, or at BAL that demonstrates an eosinophilic count greater than 25% [287,288]. Most cases of AEP are idiopathic; however, the association with exposure to cigarette smoke or drugs has been described [288]. In the *chronic* setting, *eosinophilic pneumonia* (CEP) maintains elevated peripheral blood eosinophils, and nonsegmental areas of airspace consolidation with peripheral upper lobe predominance are present on CT [289]. A rare, chronic entity with increased eosinophils is *Churg–Strauss syndrome*; it is an eosinophilic vasculitis characterized by hypereosinophilia, asthma, paranasal sinus abnormalities, and bilateral peripheral opacities. However, unlike CEP, in this case, consolidations tend to have lobular distribution associated with centrilobular nodules [288].

*HP* could have an acute, subacute, or chronic presentation; it is observed in non-smoker patients with a history of inhalation of specific organic antigens. Most cases occur following months or years of continuous or intermittent inhalation of the inciting agent [237]. In acute onset, it demonstrates normal lung regions mixed with patchy GGO. In the chronic setting, there is the onset of fibrotic signs and air trapping, as previously mentioned. This pattern is often described as the “three-density pattern” (or “headcheese sign”). Bronchiolar wall thickening and mediastinal lymph node enlargement are frequently associated [238].

*Desquamative interstitial pneumonia* (DIP) is typical for heavy smokers and can be considered the end spectrum of RB-ILD. The presence of peripheral and subpleural diffuse GGO with basal predominance reflects the alveolar accumulation of pigmented macrophages [219]. Unlike in RB-ILD, functional tests show a marked reduction in diffusion capacity and a moderate restrictive pattern, which results in severe dyspnea and stress hypoxemia [290].

Although rare, pulmonary alveolar proteinosis (PAP) is worth a short mention, since it classically demonstrates clinical–radiologic discrepancy where the patient does not demonstrate relevant symptoms but extensive bilateral, central, and symmetric GGO with superimposed smooth interlobular septal thickening (“crazy paving”) are present [291]. This is a mostly idiopathic condition, and definitive diagnosis requires lung biopsy or BAL specimens which demonstrate intra-alveolar deposits of proteinaceous material [292].

##### Consolidations

On CT, a consolidation is a homogeneous increase in the pulmonary density that obscures the airway walls and vascular structures; it is the consequence of an exudate or other materials in several pathological processes [79]. For the differential diagnosis of consolidations, the acute or chronic condition, together with the distribution and ancillary findings, are helpful for the characterization.

In an acute setting, the most frequent diseases to be considered are infectious pneumonia and aspiration. Among infections, viral pneumonia, atypical bacterial pneumonia (e.g., legionella, mycoplasma, and chlamydia), and PJP usually present with a diffuse pattern, but a GGO pattern is more common in these conditions. Conversely, aspiration, hemorrhage, and other infections (i.e., typical bacterial, fungal, or mycobacterial) have a typical focal distribution. Other causes of consolidation in the acute setting are pulmonary oedema and diffuse alveolar damage. These conditions usually have a diffuse distribution; however, GGOs are more typical findings than consolidations in these conditions.

In a chronic setting, the distribution of consolidations is helpful to narrow the differential diagnosis to a few pathological conditions. The most frequent conditions associated to chronic consolidations are OP, chronic eosinophilic pneumonia, sarcoidosis, invasive mucinous carcinoma, lymphoma, and hypersensitivity pneumonitis. Considering that all chronic conditions may present a patchy distribution, invasive mucinous carcinoma has a focal or diffuse presentation, sarcoidosis or OP have a peribronchovascular distribution, while chronic eosinophilic pneumonia or OP can have a peripheral distribution. Lipoid pneumonia is a rare cause of focal, hypodense consolidations (typically < 30 HU). It is the result of aspiration of fat-containing fluids and is often asymptomatic [293,294].

As previously mentioned, the reversed-halo sign is an accessory finding for the diagnosis of OP. Accessory findings for the diagnosis of sarcoidosis are symmetrical, enlarged hilar lymph nodes and the galaxy sign, which is composed of multiple small, confluent nodules close to the margins of a larger consolidation [234].

#### 4.2.4. Decreased Lung Attenuation

For diseases with subpleural cystic spaces (honeycombing and paraseptal emphysema), please refer to the respective section above.

The most recognizable feature with decreased lung attenuation in smoker patients is *centrilobular emphysema* (CLE). It is characterized by destroyed centrilobular alveolar walls and enlargement of respiratory bronchioles and associated alveoli. It demonstrates highly characteristically centrilobular air-attenuation cystic lucencies, usually smaller than 1 cm, without a visible wall [79].

Conversely, a cyst is defined as any round circumscribed space that is surrounded by an epithelial or fibrous wall of variable thickness [79]. When multiple cysts are discovered in a young smoker patient (age 20–40), consider *Langherans’ cell histiocytosis* (LCH). LCH symptoms vary widely, from asymptomatic to severe dyspnea; the initial clinical presentation may be with a pneumothorax. The key findings are cystic lesions (usually <10 mm) with a thin or thick wall that may coalesce assuming bizarre and irregular shapes with an upper lobe predominance and sparing of basal costophrenic angles [295].

Other cystic lung diseases are even rarer; however, *lymphangioleiomyomatosis* (LAM) demonstrates characteristic findings at imaging that allow an easy diagnosis. LAM is a rare multisystemic disorder characterized by the proliferation of abnormal smooth muscle-like cells in the walls of airways, venules, and along the axial lymphatic system, leading to progressive cystic lung destruction. LAM characteristically almost exclusively affects young women of childbearing age (20–40 years); key features are rounded thin-walled cysts (2–60 mm) with uniform shape, and bilateral involvement of the lung from apex to base. A few scattered cysts may be present or there may be near-complete replacement of the lungs without sparing the costophrenic angles. In 50% of cases, the primary manifestation is pneumothorax. Extrathoracic-related findings are present in >70% of cases as retroperitoneal lymphadenopathies, the presence of multiple abdominal angiomyolipomas, and chylous ascites [296]. Similarly, LIP presents with bilateral, thin-walled cysts causing pneumothorax. However, in this last condition, the cysts are fewer in number with perivascular or subpleural distribution, and a history of connective tissue disease or immunosuppression can be present [209].

## 5. Conclusions

Facing chest pathologies, radiologists must recognize a wide range of different entities, from the mediastinum to pleuro-pulmonary involvement.

Some have typical imaging features leading to an immediate diagnosis; others are very difficult to identify with the sole evaluation of radiologic findings. However, knowledge of basic imaging findings, combined with clinical data, is helpful in most cases.

## Figures and Tables

**Table 1:** International Thymic Malignancy Interest Group (ITMIG) classification of mediastinal compartments [9].

Compartment	Boundaries	Content	PA Chest X-ray Sign
**Prevascular (anterior)**	Anterior: posterior cortex of the sternum (mammary vessels excluded). Posterior: anterior aspect of the pericardium.	Thymus, mediastinal fat, lymph nodes, the left brachiocephalic vein.	Hilum overlay sign. Anterior junction line not visible. Preservation of the posterior mediastinal lines. If located over the level of the clavicles: not sharp margins.
**Visceral (middle)**	Anterior: posterior boundaries of the prevascular compartment. Posterior: a plane 1 cm beyond the anterior aspect of the vertebral bodies.	Nonvascular: pericardium, trachea, esophagus, lymph nodes. Vascular: heart, superior vena cava, ascending and descending thoracic aorta, thoracic duct, and intra-pericardial pulmonary arteries.	Widening or obliteration of the right paratracheal stripe. Widening of the aortopulmonary window.
**Paravertebral (posterior)**	Anterior: posterior boundaries of the visceral compartment. Posterolateral: a plane along the posterior margin of the chest wall at the lateral margin of the transverse processes.	Thoracic spine. Paravertebral soft tissues.	Hilum overlay sign. Deviation or disruption of the azygoesophageal line or paraspinal lines. If located superior to the level of the aortic arch: the obliteration of the posterior junction line. If located above the level of the clavicles: sharp margins.

**Table 2:** Mediastinal masses: differential diagnosis by location and attenuation.

Compartment	Fat Attenuation	Cystic Component	Soft-Tissue-Enhancing Masses
**Anterior** **(prevascular)** **(69.8%)**	(*8.4%*): Thymolipoma Lipoma Liposarcoma Epicardial fat pad Morgagni hernia	(*24%*): Pericardial cyst Cystic thymoma Lymphangioma Cystic teratoma	Thyroid goiter * Thymoma (*30.8%*) * Thymic carcinoma (*7.5%*) * Lymphoma (*14.4%*) Mature teratoma * Thymic hyperplasia Parathyroid adenomas *
**Middle** **(visceral)** **(13.5%)**		Bronchogenic cyst (*16.8%*) *	Thyroid goiter (*13%*) * Lymphadenopathy/metastasis (*22.4%*) Esophageal cancer
**Posterior** **(paravertebral)** **(5.4%)**	Extramedullary hematopoiesis	(*13.9%*): Lateral meningocele Pseudocyst	Neurogenic neoplasms (*53.9%*)
**>1 compartment** **(11.2%)**	Liposarcoma Lipomatosis	Lymphangioma	Lymphadenopathy Lung cancer

Legend. (*) the lesion may contain calcifications. The frequencies of the mediastinal lesions for each compartment (expressed in percentages and in bold) in the first column (i.e., “compartment”) refer to the total population included in Roden et al. [19]. The frequencies (expressed in percentages and in italics) of the most frequent lesion or lesion category in the second, third, and fourth columns (i.e., fat, cystic, or softtissue-enhancing masses) refer to the total lesions for each compartment (i.e., the row) in Roden et al. [19].

**Table 3:** General features of the likelihood of benignity or malignity of a solitary pulmonary nodule (SPN).

	Benignity	Malignancy
**Size**	<6 mm	>3 cm
**Volume doubling time (VDT)**	VDT of <30 days or >400 days	VDT between 30 and 400 days
**Margin**	Smooth, rounded	Irregular, lobulated, or spiculated
**Density**	Fat	
	Calcification (Central laminated, popcorn, and diffuse patterns of calcification)	Calcification (Punctate, eccentric patterns of calcification)
	Air (thin and regular walls, <5 mm)	Air (thick, irregular walls, <15 mm)
**Shape**	Round lesions with smooth margin	Irregular shape with spiculated or lobulated margin, pleural tags
	Triangular lesions in subpleural and perifissural locations	
	Polygonal, elongated, elliptical, linear, or plaque-like shape	
**Location**	Perifissural nodules, predominately represent perifissural lymph nodes	Upper lobe distribution is associated with an increased risk of malignancy with an odds ratio of 1.9

Legend: VDT: volume doubling time. Period ranges are from reference [143].

**Table 4:** Differential diagnostic features for paraseptal emphysema and honeycombing.

	Paraseptal Emphysema	Honeycombing
**Layers**	Always one layer	One or more layers
**Wall Thickness**	Very thin	Thick
**Associated findings**	Centrilobular emphysema	Traction bronchiectasis
**Distribution**	Upper lobes	Lower lobes
**Size**	Large	Small
**Overall lung volume**	Increased	Decreased
**Associated reticulation**	Absent	Present

## Data Availability

Not applicable.
